# Atypical anterior persistent hyperplastic primary vitreous: report of a rare case

**DOI:** 10.1186/s12886-020-01539-1

**Published:** 2020-07-16

**Authors:** Jue Wang, Hong Yan, Zhaojiang Du, Jie Zhang, Weinong Wang, Chenjun Guo

**Affiliations:** 1 Department of Ophthalmology, Tangdu Hospital, Fourth Military Medical University, No. 1, Xinsi Road, Xi’an, Shaanxi 710038 People’s Republic of China; 2 Xi’an No. 4 Hospital, Shaanxi Eye Hospital, ffiliated Hospital of Northwestern Polytechnical University, Xi’an, Shaanxi People’s Republic of China; 3 Xi’an Central Hospital, Xi’an, Shaanxi People’s Republic of China

**Keywords:** Persistent hyperplastic primary vitreous, Acute glaucoma, Atypical anterior PHPV, Surgery, Case report

## Abstract

**Background:**

Persistent hyperplastic primary vitreous (PHPV) is a congenital form of vitreous dysplasia that can be categorized into anterior, posterior, and mixed types according to the affected location within the eye. Definitive diagnoses of PHPV are usually made based on B-mode ultrasound, optical coherence tomography (OCT), and Doppler ultrasound findings. In this report, we discuss the case of a 7-year-old boy in whom a definitive diagnosis of atypical anterior PHPV was possible based on intraoperative observations, pathological findings, and the results of ophthalmic examination.

**Case presentation:**

A 7-year-old boy presented with leukocoria and acute glaucoma in his right eye. Imaging suggested characteristics of mixed PHPV. Surgical treatment and pathological examination were performed due to the presence of acute glaucoma and abnormal lens morphology. Typical signs of posterior PHPV (e.g., eyeball shrinkage, the presence of vascular membranes connected to the optic disc, etc.) were not observed. However, there were abundant fibrous vascular membranes around the lens. Pathological examination revealed fibrocyte proliferation in the lens and capsular tissue. Intraoperative findings were used in conjunction with the results of pathological and ophthalmological examinations to make the final diagnosis of anterior PHPV.

**Conclusion:**

The course and characteristics of PHPV can be unpredictable, and it is often the case that a clear diagnosis cannot be obtained based on clinical characteristics and typical imaging examinations alone. Further surgical treatment and pathological examination may aid in establishing a final diagnosis. In addition to treating the complications of PHPV (e.g., glaucoma), surgery may improve eye appearance and restore visual function to some degree.

## Background

Persistent hyperplastic primary vitreous (PHPV), also known as persistent fetal vasculature (PFV), is a congenital form of ocular dysplasia that occurs secondary to failure of normal vitreous development or persistent proliferation of the original vitreous during the embryonic period [1]. Clinical symptoms include small globes, small corneas, ciliary body elongation towards the center, posterior fibrous membranes within the lens, and persistent vitreous arteries.

PHPV can be divided into three types, based on the affected ocular structures [2]. Anterior PHPV (approximately 25% of cases) is associated with lens opacity or posterior capsule cortical opacity, continuous fibrovascular membrane hyperplasia behind the lens, and elongation of the ciliary body. Posterior PHPV (approximately 12% of cases) is associated with connections between the vitreous vascular membrane and the optic disc, eyeball shrinkage, and immature growth of the optic disc, macula, and retina. In mixed PHPV (approximately 63% of cases), both anterior and posterior signs can be observed.

Complications of PHPV include angle-closure glaucoma, vitreous hemorrhage, and retinal detachment. PHPV is also among the common causes of infantile leukocoria and is easily misdiagnosed as congenital cataract [3]. Diagnoses of PHPV are typically made based on the results of B-mode ultrasound, optical coherence tomography (OCT), or Doppler ultrasound [4, 5]. Although it remains controversial, surgical treatment of PHPV may improve eye appearance, aid in the recovery of visual function, and help clinicians diagnose ocular diseases.

In this report, we discuss the case of a 7-year-old boy in whom imaging failed to yield a clear diagnosis. However, he exhibited acute glaucoma necessitating surgery. Following surgery, a clear diagnosis of PHPV was established based on intraoperative observations, pathological findings, and the results of ophthalmic examination.

## Case presentation

**Fig. 1:**
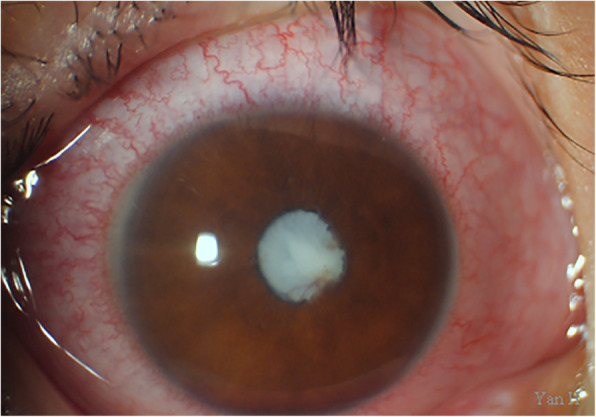
Anterior section image obtained at the initial visit. The image shows conjunctival mixed hyperemia, shallowness of the anterior chamber, posterior synechia of the iris, a pupil diameter of 2 mm, and a cataractous lens

**Fig. 2:**
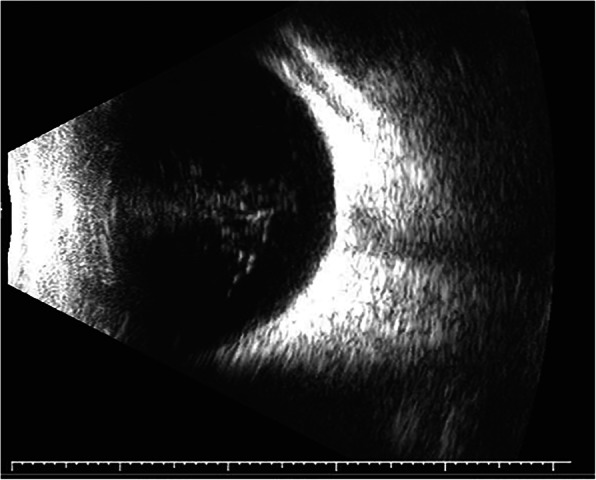
B-mode ultrasound image obtained at the initial visit. The image shows a wide, turbid band extending from the back of the lens to the front of the optic disc. Axis length in the right eye was 26.32 mm

A 7-year-old boy was admitted to our hospital in April 2019 due to whitening of the pupil area in the right eye after a fall, which was accompanied by eye pain, headache, and vomiting for 3 days. Best corrected visual acuity (BCVA) was 1.0 in the left eye. However, only the perception of light was possible in the right eye. The right eye also exhibited mixed conjunctival hyperemia (+++), mild corneal edema, an absence of wrinkles in the endothelium, a shallow anterior chamber, an irregular pupil shape (diameter: 2 mm), an absence of the light reflex, a rough lens surface, and pigment adhesion (Fig. 1). Intraocular pressure in the right eye was 42.3 mmHg, while that in the left eye was normal. Upon admission to our hospital, he was given a preliminary diagnosis of traumatic cataract with secondary glaucoma of the right eye. However, during his hospitalization, we learned that he had been diagnosed with congenital cataract of the right eye at a local hospital 7 years earlier. Therefore, the diagnosis was revised to glaucoma secondary to congenital cataract of the right eye. Treatment with intraocular pressure-lowering drugs proved effective. However, B-mode ultrasound examination revealed a wide echogenic band extending from the back of the lens to the front of the optic disc in the right vitreous cavity (Fig. 2). Axis length for the right eye was 26.32 mm, while that for the left eye was 22.30 mm. Ultrasound biomicroscopy (UBM) revealed an anterior chamber depth of 1.05 mm in the right eye. UBM also indicated upward bulging of the right iris at 3–7 points. The angle had closed, the posterior chamber was no longer visible, and the anterior capsule echo of the lens was irregular. Analysis of flash visual-evoked potentials (F-VEP) revealed delayed P2 and P3 peaks in the right eye, relative to those in the left eye. Given that B-mode ultrasonography also revealed fibrous vascular membranes around the lens, the diagnosis was again revised to congenital cataract, PHPV, and secondary glaucoma of the right eye.

**Fig. 3:**
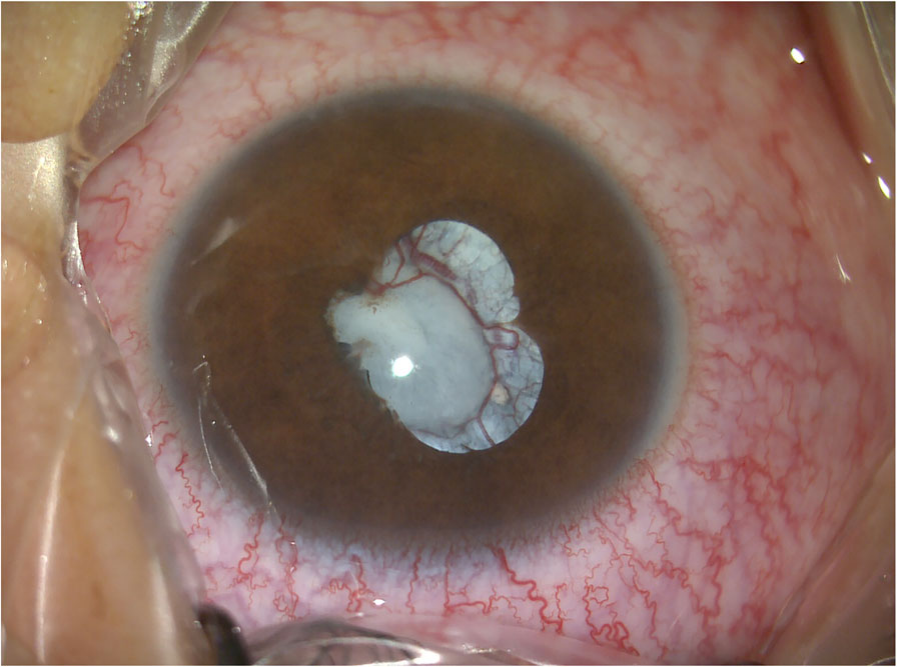
Anterior section image obtained prior to surgery. The diameter of the dilated pupil was 5 mm, and the pupil exhibited an irregular shape. We also observed posterior synechia of the iris and massive integration of vascular tissue within the lens

**Fig. 4:**
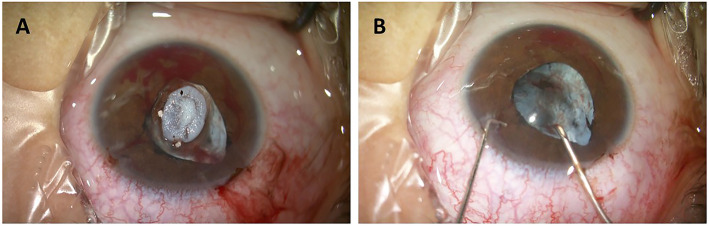
Observation of lens morphology during surgery. The image shows a white, wrinkled lens. The diameter of the detaching lens was approximately 3 mm (**a**). The posterior capsule is also shown (**b**)

Surgery was indicated due to the presence of acute glaucoma and abnormal lens morphology. Cataract extraction and anterior vitrectomy were thus performed under general anesthesia. Prior to the operation, the diameter of the dilated pupil was 5 mm, and the pupil was irregular in shape. In addition, we observed posterior synechia of the iris and massive vascular tissue integration within the lens (Fig. 3). During surgery, after detaching the posterior synechia of the iris, we noticed deficiencies in the anterior capsule of the lens. The lens appeared wrinkled and had a diameter of approximately 3 mm (Fig. 4). Substantial integration of vascular tissue was observed in the posterior capsule of the cataractous lens. Following posterior capsulotomy, the red light reflex disappeared. We then performed anterior vitrectomy.

**Fig. 5:**
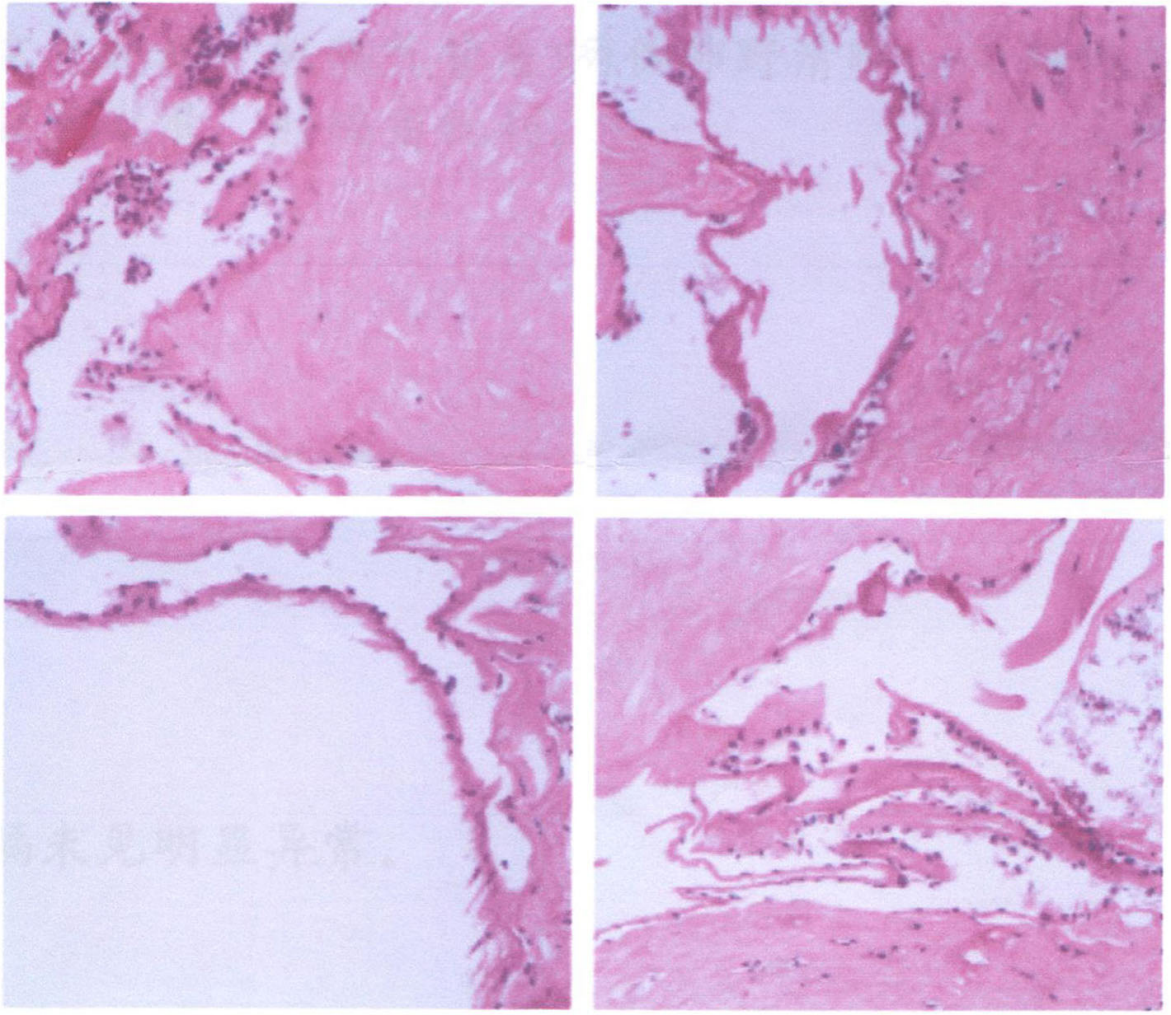
Postoperative pathological staining of the lens tissue. Four fields of view were selected. Pathological examination revealed that the right eye lens and capsule contained little connective tissue. Fibrous tissue hyperplasia, hyaline degeneration, calcification/follicular cystic changes, and active fibrocyte proliferation were also observed

Pathological examination revealed that the right lens and capsule contained little connective tissue. We also observed fibrous tissue hyperplasia, hyaline degeneration, calcification, and follicular cystic changes, in addition to active proliferation of fibrocytes (Fig. 5).

**Fig. 6:**
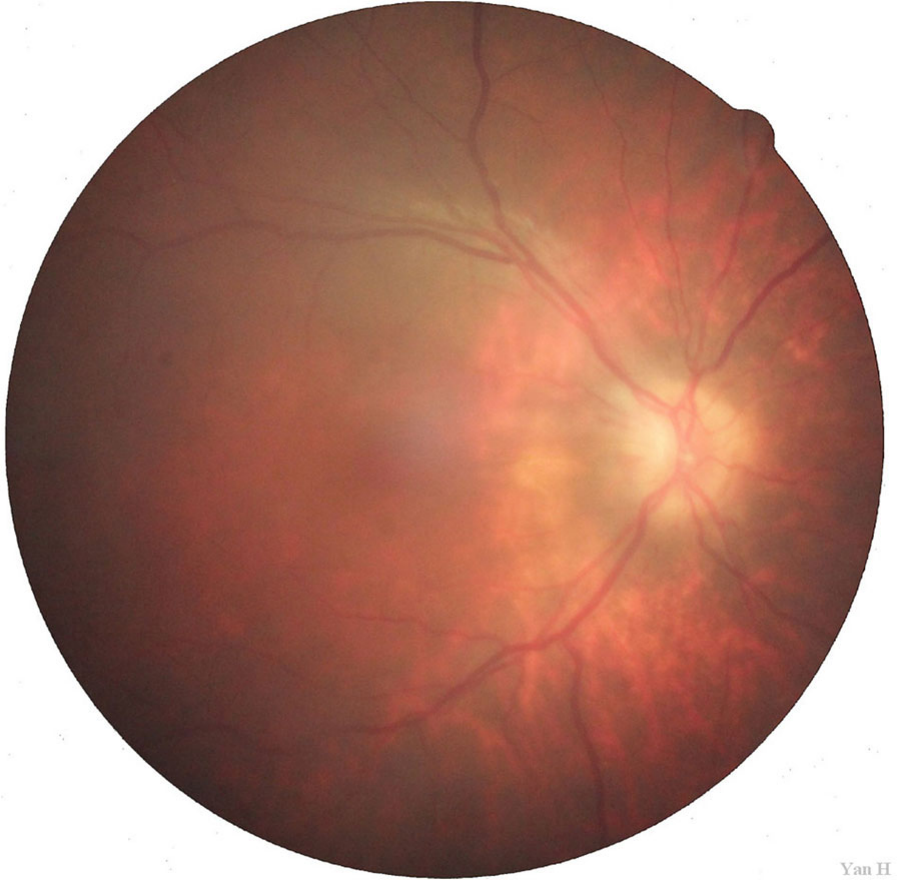
Fundus examination after surgery. The boundary of the right optic disc was blurred. The cup-to-disk (C/D) ratio was approximately 0.1, and the retina exhibited applanation. The blood vessels were well-shaped

BCVA remained unchanged in either eye following surgery. Postoperative examination further revealed a transparent right cornea. The right anterior chamber was deep, the pupil was round, pupil diameter was 3 mm, the lens was absent, and mild vitreous opacity was observed. Fundus examination revealed blurring of the boundary with the right optic disc. The cup-to-disk (C/D) ratio was approximately 0.1, and the retina exhibited applanation. The blood vessels were well-shaped, and the macular structure was generally normal (Fig. 6). Optic OCT indicated that the thickness of the retinal nerve fiber layer (RNFL) was 0 μm, although the macular structure of the right eye was normal. Intraocular pressure was 17.3 mmHg in the right eye.

Neither intraoperative nor postoperative examinations revealed characteristics of PHPV such as connection of the vitreous fibrous vascular membrane to the optic disc, shrinkage of the eyeball, or retinal dysplasia. Furthermore, the lens and capsule were severely fibrotic, with an abundance of surrounding vascular tissue. The final diagnosis was anterior PHPV with secondary glaucoma in the right eye. Although further visual rehabilitation training was recommended, his parents declined. At the 6-month follow-up, visual acuity in the right eye had not improved, and findings in the remaining anterior and posterior segments were identical to those of the postoperative examination.

## Discussion and conclusions

Pathological features of anterior PHPV include proliferation of the posterior fibrous vascular membrane, such that the membrane eventually covers the posterior surface of the lens and invades the ciliary process. Proliferation and contraction of the posterior fibrous membrane of the lens alters the structure of the anterior segment. The ciliary body is pulled toward the center, with some patients exhibiting elongation of the ciliary process following dilation [6]. The fibrous vascular membrane also begins to cover the posterior capsule of the lens, eventually growing into the lens itself, which may lead to spontaneous hemorrhage within the lens. As the tension increases, the posterior capsule of the lens becomes more likely to rupture, which leads to rapid cataract formation. Following inflation, the iris diaphragm of the lens is pushed forward, and the anterior chamber becomes shallower or even disappears, resulting in secondary glaucoma. As the anterior chamber becomes shallower, extensive uveal reactions such as posterior synechia and peripheral anterior synechia may eventually lead to corneal opacification [7–9].

Previously, many researchers and clinicians believed that surgical treatment for PHPV rarely resulted in good prognosis. Indeed, conservative treatment can maintain the stability of vision and is associated with fewer complications than surgical treatment. The only absolute indication for surgery in patients with PHPV is intractable high intraocular pressure [10, 11]. However, recent evidence indicates that the prognosis of surgical intervention is related to the time window of visual development in children. In young children, surgical treatment followed by refractive correction and treatment for amblyopia is very likely to restore vision to some degree [12, 13]. Even in some adult patients, vitrectomy and crystallography may also lead to exponential improvements in vision. Advancements in microsurgical techniques have improved the ability to restore appearance and visual function while reducing complications in patients with PHPV. Kanigowska et al. [14] reported the case of a 3-month-old child with mixed PHPV and secondary glaucoma due to pupillary block, which was caused by total dislocation of the lens. After surgery, the intraocular pressure returned to normal, creating the conditions for recovery of visual function. Brennan et al. [15] further noted slight improvements in vision following surgery in an adult patient with PHPV who had secondary glaucoma due to posterior capsule rupture.

In our patient, surgery was indicated due to the presence of acute glaucoma and abnormal lens morphology. However, our case is unique in that all previously mentioned reports included patients with mixed PHPV rather than anterior PHPV. Although the diagnosis required several revisions, surgical treatment played a decisive role in determining the final diagnosis. Given our patient’s age (7 years), we wish to further emphasize the positive impact that surgical treatment for severe cataracts can exert on visual function and eye appearance.

Although the lens opacity and posterior fiber vascular membrane hyperplasia observed in our patient were consistent with the diagnosis of anterior PHPV, some atypical features were also noted. B-mode ultrasound of the right eye revealed turbidity of the intravitreal cavity extending from the back of the lens to the front of the optic disc, with no vitreous vascular membrane connecting the optic disc to the posterior lens. We speculate that the original vitreous degradation occurred during the embryonic period, leaving behind the vitreous turbidity. Furthermore, the globes are typically small in patients with PHPV. In contrast, eyeball diameter was longer in the affected eye than in the normal eye in our patient, which may have been caused by long-term increases in intraocular pressure. Our patient also exhibited abnormal development of the optic disc after the operation. Although this is consistent with the characteristics of optic disc/retinal hypoplasia in patients with posterior PHPV, our patient also had an RNFL thickness of 0 in the optic disc. This finding suggests that sustained increases in intraocular pressure led to compression of the optic disc.

The course and characteristics of PHPV can be unpredictable, and it is often the case that a clear diagnosis cannot be obtained based on clinical characteristics and typical imaging examinations alone. Further surgical treatment and pathological examination may aid in establishing a final diagnosis. In addition to treating the complications of PHPV (e.g., glaucoma), surgery may also improve eye appearance and restore visual function to some degree.

## Data Availability

All data supporting the findings are contained within the manuscript.

## Abbreviations

PHPV: Persistent hyperplastic primary vitreous
BCVA: Best corrected visual acuity
PFV: Persistent fetal vasculature
OCT: Optical coherence tomography
UBM: Ultrasound biomicroscopy
F-VEP: Flash-visual evoked potentials
C/D: Cup-to-disk ratio
RNFL: Retinal nerve fiber layer
